# Cannabis Indica in Colic*Read before the Nebraska Veterinary Medical Association.

**Published:** 1891-05

**Authors:** S. Stewart


### CANNABIS INDICA IN COLIC.*

By S. Stewart, M. D., D. V. M.

This drug is found in the shops in the form of coarse powder, tincture, fluid extract and solid extract. It has long been used by human physicians for the relief of spasm and pain. It finds an occasional mention in veterinary literature. Finlay Dunn says little concerning it; classes it as narcotic, anodyne, and anti-spasmodic; and claims that it has no influence over normal respiration, circulation or temperature. He quotes Sir Robert Christianson as stating that " he has long been convinced that for energy, certainty and convenience, Indian hemp is the next anodyne, hypnotic and anti-spasmodic to opium and often equals it."

Doctor H. C. Wood once took a large dose of the infusion of hemp and knows from his experience that it obtunds the motor nerves as well as the sensory, and produces a peculiar disturbance of intellection, distorting both the sense of distance and duration.

Negative reports concerning the action of this drug have frequently been made because inert products have been supplied to the profession, but manufacturing pharmacists now produce more trustworthy preparations.

The fluid extract is the most reliable form of the drug and is a very convenient form to administer. Experience has taught me

* Read before the Nebraska Veterinary Medical Association.

that the mucous membrane of the mouth is an excellent absorbing organ and more trustworthy than the stomach, and I give this drug in one or two fluid dram doses, undiluted, by pouring it on the tongue from a small bottle or by injecting it into the mouth with a syringe. Oftentimes a single dose is sufficient, if not, it should be repeated in fifteen or twenty minutes, or more, and as often as the exigencies of the case require. I have given sufficient of the drug to produce hallucinations, yet no unpleasant after effects were noticed. It is a safe drug to leave in the hands of attendants for administration.

I have used this drug in about fifty cases and it has not failed to do what I expected it to do, namely, relieve spasms and abdominal pain. I have discarded opium and chloral hydrate in this class of cases, and do not expect to resort to them so long as this agent serves me so faithfully. In simple spasmodic colic no other medicine is needed. There are many cases of spasmodic colic which recover without treatment, and many cases which seem to yield to any plan of treatment which would probably have recovered spontaneously if not interfered with. Yet many cases do not recover even when drugs and manipulations are resorted to. When given charge of a case of spasmodic colic, the veterinarian cannot foretell the results, and seldom depends upon unaided nature to affect a cure. I give these cases cannabis indica; they recover.

In cases of acute indigestion, cannabis indica will relieve the pain, while other mendicaments correct the digestive disturbance. The same thing is true in obstruction or impaction of the colon.

A fine roadster, four years old, which was running in pasture daytimes and was stabled nights, during the Fall of 1890, contracted spasmodic colic one stormy night and was turned into an enclosed wagon-room while a surgeon could be summoned. Upon arrival at the farmer's barn some four miles from town, I found the colt rolling and tumbling violently; the hair and cuticle had been abraded from prominent portions of his face, giving the horse a dilapidated appearance; the paroxysms of pain recurred at short intervals; the temperature of rectum was normal, the attack had not been preceded by diarrhœa or scouring, the abdomen was not tympanitic. Two-dram doses of fluid extract cannabis indica were given every fifteen minutes for an hour, at which time decided relief was obtained; one-half hour later, the horse was dozing from narcosis produced by this drug. The attendant was instructed to give two-dram doses of the drug every two hours during the remainder of the day. When the case was visited the next morning, the attendant reported that the pain had not returned but that the colt had stood very quietly and apparently had slept most of the time. Urine was passed easily and abundantly, no fæces were discharged, no food was taken. Found pulse 50, respiration 16, temperature 102. Instructed attendant to give the drug in the same dose every four hours until further notice. On the morning of the second day I found the temperature normal, respiration 12, pulse 44. Stalling had been profuse and a small quantity of fæcal matter had been discharged, the appetite was good. Convalescence was rapid without further medication.

A 1,200 pound work-horse used for hauling dirt was brought to my hospital at 5 P. M., he had eaten the noon ration as usual and showed no signs of illness until about 4 P. M. The horse gave evidence of spasmodic pain in the intestinal tract and was slightly tympanitic. Thinking this case depended upon intestinal indigestion, I gave one ounce of turpentine with one quart of linseed oil, followed by four or five two-dram doses of cannabis indica. The last drug subdued the pain and the oils cleared away the irritating ingesta. The next morning this horse called for food and required no further medication.

I have given this drug in the same manner to cows suffering colicky pains with just as gratifying results.

The details of many cases could be given if I thought they would interest you but I refrain from so doing, knowing that a trial of the drug will do more to convince you than all the cases I could describe. I would have you to understand that I do not recommend cannabis indica as a panacea for all cases of colic, or that it is to be depended upon to the exclusion of all other medicaments; but I do say that I have found it a certain agent for the relief of pain attendant upon bowel disturbances, that it does not produce delirium and constipation like opiates, that it does not blister the mouth like chloral, that it is always ready and easy of administration and a safe drug to leave for attendants to administer.

